# Rethinking Risk Prediction in Preeclampsia: From Biomarkers to Mechanistic Phenotypes and Longitudinal Models

**DOI:** 10.3390/ijms27083480

**Published:** 2026-04-13

**Authors:** Salvador Espino-y-Sosa, Elsa Romelia Moreno-Verduzco, Irma Eloisa Monroy-Muñoz, Juan Mario Solis-Paredes, Javier Pérez Durán, Lourdes Rojas Zepeda, Johnatan Torres-Torres

**Affiliations:** 1 Department of Bioinformatics, Instituto Nacional de Perinatologia Isidro Espinosa de los Reyes, Mexico City 11000, Mexico; salvadorespino@gmail.com (S.E.-y.-S.); juan.mario.sp@gmail.com (J.M.S.-P.); 2 Department of Reproductive and Perinatal Health Research, Instituto Nacional de Perinatologia Isidro Espinosa de los Reyes, Mexico City 11000, Mexico; elsa.moreno.verduzco@gmail.com (E.R.M.-V.); irmae4901@gmail.com (I.E.M.-M.); djavier40@gmail.com (J.P.D.); 3 Maternal-Fetal Deparment, Instituto Materno Infantil del Estado de Mexico, Toluca 50170, Mexico; dra.rojaszepeda@gmail.com

**Keywords:** preeclampsia, biomarkers, risk assessment, pregnancy complications, machine learning

## Abstract

Preeclampsia remains a major cause of maternal and perinatal morbidity and mortality worldwide, yet progress in biomarker discovery and predictive modeling has translated only modestly into clinically meaningful risk stratification. Over the past two decades, numerous biomarkers and predictors reflecting placental–angiogenic dysfunction, maternal cardiovascular maladaptation, and inflammatory–metabolic stress have been proposed, alongside increasingly sophisticated statistical and machine learning approaches. However, many predictive strategies continue to treat preeclampsia as a single disease entity and rely on static thresholds applied at isolated gestational time points. Accumulating biological and clinical evidence instead suggests that preeclampsia represents a heterogeneous syndrome composed of partially overlapping mechanistic phenotypes whose relative contributions vary across pregnancy and across individuals. In this narrative review, we argue that further progress in prediction is likely to depend less on the identification of additional biomarkers and more on how biological heterogeneity and temporal dynamics are integrated into predictive frameworks. We synthesize current evidence supporting multimarker approaches, phenotype-informed frameworks, and longitudinal risk trajectories that conceptualize prediction as a dynamic process rather than a binary classification task. We also examine the complementary roles of classical statistical models and machine learning, emphasizing that calibration, external validation, interpretability, transportability, and clinical usability are essential, alongside discrimination, for successful clinical implementation. Finally, we outline key research priorities for the next generation of predictive studies, including mechanistically grounded phenotyping, dynamic risk updating across gestation, rigorous evaluation across diverse populations, and explicit linkage of risk stratification to preventive interventions and clinical decision-making. Together, these directions support a shift toward an integrative, longitudinal, and clinically anchored approach to preeclampsia prediction.

## 1. Introduction

Preeclampsia remains a leading cause of maternal and perinatal morbidity and mortality worldwide [1,2]. Despite substantial advances in understanding its pathophysiology and the identification of numerous biomarkers and clinical risk factors, early prediction and effective risk stratification in routine clinical practice remain limited [3,4]. This disconnect between scientific progress and clinical impact has renewed interest in biomarker-based prediction and advanced modeling strategies [5].

Over the past two decades, a wide range of biomarkers reflecting placental dysfunction, angiogenic imbalance, inflammation, and maternal cardiovascular maladaptation have been proposed for the prediction of preeclampsia, alongside increasingly sophisticated statistical and machine learning-based models [6,7,8,9]. Although many of these approaches demonstrate acceptable discriminatory performance in selected cohorts, their translation into clinically robust and generalizable tools has been modest. This apparent paradox suggests that the primary challenge lies not in the availability of biomarkers or analytical techniques, but in how biological information is conceptualized and integrated within predictive frameworks [5,7,8].

A central limitation of many existing strategies is the implicit assumption that preeclampsia represents a single disease entity [10,11]. Accumulating evidence instead indicates that preeclampsia is a heterogeneous syndrome comprising distinct mechanistic phenotypes that differ in underlying biology, timing of onset, clinical presentation, and associated maternal and perinatal risks [10,11,12]. Prediction approaches based on single biomarkers or static thresholds, without accounting for this heterogeneity or for the dynamic evolution of risk across gestation, are therefore inherently constrained.

Recent work integrating multiple physiopathologic biomarker domains has highlighted the potential of multimarker strategies for early risk classification [8,13]. At the same time, advances in machine learning have expanded analytical possibilities while drawing attention to challenges related to interpretability, external validity, and clinical usability [7,8,14]. In this review, we examine current approaches to preeclampsia prediction through the lens of biological heterogeneity and longitudinal modeling, and discuss how integrative frameworks may enhance clinical relevance and inform future research.

## 2. Scope and Approach

This article presents a narrative, conceptually driven review of current approaches to preeclampsia prediction, focusing on the integration of biomarkers, mechanistic phenotypes, and longitudinal predictive frameworks. The discussion emphasizes key conceptual advances and representative studies that illustrate the strengths and limitations of existing strategies.

The literature considered in this review was identified through a targeted search of major biomedical databases, including PubMed and Scopus, with a primary focus on studies published over the past two decades. Study selection was guided by relevance to key themes addressed in this manuscript, including biological heterogeneity, biomarker integration, longitudinal risk modeling, and predictive model evaluation. Priority was given to influential clinical, translational, and methodological studies rather than to exhaustive coverage.

As a narrative and conceptually driven review, this article does not aim to provide a systematic synthesis of the literature. Instead, study selection was guided by conceptual relevance to the proposed framework, and may therefore not encompass all available evidence on the topic.

## 3. Preeclampsia as a Heterogeneous Syndrome

**Figure 1:**
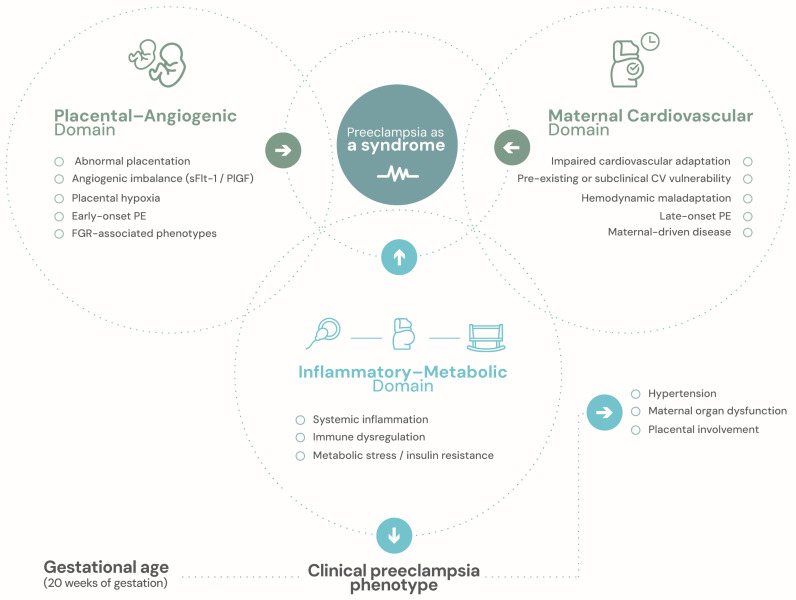
Mechanistic heterogeneity of preeclampsia and convergent clinical expression. This figure represents a conceptual schematic. Preeclampsia is best understood as a heterogeneous syndrome arising from partially overlapping mechanistic domains rather than a single disease entity. Placental–angiogenic dysfunction, maternal cardiovascular maladaptation, and inflammatory–metabolic processes contribute variably across gestation, may coexist to differing degrees, and evolve dynamically over time. These pathways ultimately converge on a shared clinical phenotype of preeclampsia, providing a conceptual foundation for phenotype-aware and longitudinal prediction frameworks.

Preeclampsia has traditionally been defined by a limited set of clinical criteria, most notably new-onset hypertension accompanied by maternal organ dysfunction or placental involvement after mid-gestation [3]. While this definition remains essential for diagnosis and clinical management, it obscures the substantial biological heterogeneity that underlies the syndrome. Increasing evidence suggests that preeclampsia is more accurately understood as a spectrum of pathophysiological processes that converge on a shared clinical phenotype, rather than as a set of discrete or mutually exclusive disease entities. [1,15,16] (Figure 1). These domains are best understood as conceptual and partially overlapping representations of underlying biological processes, rather than as fixed classifications.

One of the most extensively characterized mechanisms involves placental dysfunction and angiogenic imbalance. Abnormal placentation, impaired spiral artery remodeling, and placental hypoxia promote the release of antiangiogenic factors, such as soluble fms-like tyrosine kinase-1, ultimately leading to systemic maternal endothelial dysfunction [4,5,17]. This process reflects dysregulation of angiogenic signaling pathways, particularly the imbalance between pro-angiogenic (PlGF, VEGF) and anti-angiogenic factors (sFlt-1), contributing to endothelial dysfunction and disruption of the placental–maternal interface. This pathway is strongly associated with early-onset disease, fetal growth restriction, and adverse perinatal outcomes, and has informed the development of angiogenic biomarkers for prediction and short-term risk stratification [9]. Importantly, however, this biological profile is not universal, underscoring the limitations of a strictly placenta-centric view of preeclampsia [12].

A second major phenotype is characterized by predominant maternal cardiovascular maladaptation. In these cases, preeclampsia appears to arise from an insufficient maternal cardiovascular response to the hemodynamic demands of pregnancy rather than from primary placental pathology [11,18]. This phenotype has been associated with impaired vascular compliance, altered cardiac output adaptation, and endothelial dysfunction, reflecting molecular pathways involved in vascular tone regulation and subclinical cardiovascular disease. It is more commonly observed in late-onset presentations, typically in the absence of fetal growth restriction, and may reflect underlying cardiovascular vulnerability [19]. In this context, angiogenic biomarkers may remain within normal ranges or exhibit only modest alterations, highlighting the need for alternative predictive signals.

Inflammatory and metabolic pathways further contribute to disease heterogeneity. Dysregulated immune activation, systemic inflammation, insulin resistance, and metabolic stress have been implicated, particularly among women with obesity, diabetes, or other cardiometabolic risk factors [20,21]. These processes involve complex interactions between immune signaling pathways, cytokine networks, oxidative stress, and metabolic regulation, which may modulate placental function and maternal vascular responses. These mechanisms frequently interact with placental and vascular pathways, generating overlapping or mixed phenotypes rather than discrete categories [22]. As a result, rigid classifications based solely on gestational age at onset or isolated biomarkers fail to capture the biological complexity of the syndrome.

Recognition of preeclampsia as a heterogeneous condition has important implications for prediction. While distinctions such as early- versus late-onset disease or the presence of fetal growth restriction are clinically useful, they do not fully reflect underlying biology. Mechanistic phenotypes cut across these traditional categories and evolve dynamically throughout pregnancy. Prediction strategies that fail to account for this heterogeneity risk oversimplification, limited generalizability, and reduced clinical utility. These observations suggest that prediction models that do not explicitly account for mechanistic heterogeneity are unlikely to generalize reliably across populations.

Understanding preeclampsia as a syndrome composed of multiple, partially overlapping and dynamically interacting mechanistic pathways provides a necessary foundation for moving beyond single-marker approaches and motivates the development of phenotype-aware and longitudinal prediction frameworks discussed in subsequent sections (Table 1). Together, these domains reflect interconnected molecular pathways that give rise to distinct but overlapping biological phenotypes, providing a mechanistic basis for phenotype-informed prediction strategies.

## 4. Limitations of Single-Biomarker and Static Threshold Approaches

Efforts to predict preeclampsia have traditionally focused on identifying individual biomarkers capable of discriminating between women who will and will not develop disease. This strategy has yielded important biological insights and clinically useful tools, particularly for short-term risk stratification. At the same time, reliance on single biomarkers and static decision thresholds has exposed fundamental limitations that restrict predictive performance and clinical applicability.

Angiogenic markers illustrate these limitations clearly. The sFlt-1/PlGF axis reflects a central pathophysiological pathway linked to placental dysfunction and endothelial injury, and its value for ruling out imminent disease has been well established [9,23]. However, angiogenic imbalance is neither universal nor temporally stable across all forms of preeclampsia [24]. Many women who later develop preeclampsia—particularly those with late-onset presentations or predominantly maternal cardiovascular phenotypes—may exhibit normal or only modestly altered angiogenic profiles in early pregnancy, with more marked imbalance often emerging closer to clinical diagnosis [12]. Prediction strategies that rely on a single biological signal therefore risk systematic misclassification of clinically relevant subgroups.

Static thresholds further compound these challenges. Most biomarker-based approaches apply fixed cutoffs at a single time point, implicitly assuming that risk is binary and temporally invariant. In reality, preeclampsia risk evolves continuously throughout gestation, shaped by dynamic interactions among placental development, maternal adaptation, and environmental or metabolic stressors. A biomarker value that is unremarkable at one gestational age may acquire prognostic significance weeks later, while the same absolute value may reflect distinct biological states depending on timing and context. Static thresholds consequently obscure temporal patterns that are central to disease development.

Single-marker approaches also have limited capacity to accommodate biological heterogeneity. By design, they prioritize one dominant pathway while downweighting or ignoring others. This limitation is particularly relevant in preeclampsia, where overlapping mechanistic processes frequently coexist. Even when multiple biomarkers are assessed, they are often interpreted independently rather than as components of an integrated biological profile. Such fragmentation constrains the ability to capture pathway interactions and to align prediction with mechanistic phenotypes.

From a clinical standpoint, these limitations translate into modest generalizability and uncertain decision support. More broadly, the clinical value of prediction tools depends not only on discrimination but also on calibration and decision-analytic performance [25,26].

Collectively, these considerations indicate that the limited clinical impact of biomarker-based prediction in preeclampsia reflects not failure of the biomarkers themselves, but shortcomings in the conceptual frameworks in which they are applied. Moving beyond single-marker and static-threshold paradigms requires approaches that explicitly account for biological heterogeneity, temporal dynamics, and interactions among pathways—principles that underlie emerging multimarker and longitudinal prediction strategies discussed in the following section.

## 5. Multimarker and Longitudinal Risk Trajectories

**Figure 2:**
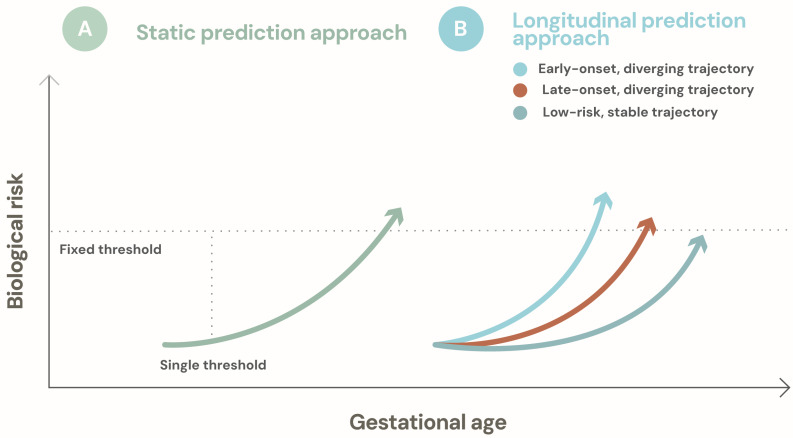
Longitudinal risk trajectories for phenotype-informed prediction of preeclampsia. This figure represents a conceptual schematic of risk evolution over time. Static prediction approaches rely on fixed thresholds applied at single time points and may fail to capture early biological divergence. In contrast, longitudinal prediction frameworks model risk as a dynamic process across gestation, allowing distinct trajectories—such as early-onset, late-onset, and low-risk patterns—to be identified over time. This trajectory-based perspective enables phenotype-informed risk stratification and supports more timely and clinically relevant prediction.

The limitations of single-biomarker and static prediction approaches have driven growing interest in multimarker strategies and longitudinal modeling frameworks for preeclampsia risk assessment. Rather than seeking a universal predictor, these approaches conceptualize risk as a dynamic process that emerges from the interaction of multiple biological pathways over time [27,28,29]. This perspective represents a fundamental shift in how prediction is framed, moving away from snapshot-based classification toward trajectory-based risk assessment (Figure 2).

Multimarker strategies aim to integrate complementary biological signals across distinct mechanistic domains, including placental development, angiogenic balance, maternal cardiovascular adaptation, and inflammatory or metabolic stress. By combining information across pathways, these approaches acknowledge that no single biomarker can capture the full complexity of preeclampsia [27,28,30,31]. Critically, the value of multimarker integration lies not in maximizing the number of measured variables, but in the deliberate selection of biologically informative markers that reflect relevant processes at specific gestational windows.

Importantly, these biomarkers are best understood not as isolated signals, but as reflections of underlying molecular pathways—including angiogenic signaling, endothelial function, and immune–metabolic regulation—that evolve over time and jointly shape disease trajectories.

The clinical implications of this dynamic perspective can be illustrated with a simple conceptual example. In a conventional first-trimester screening model, a woman with a predicted risk of preterm preeclampsia of 1:150 may fall below the threshold for preventive intervention. However, if repeated measurements of angiogenic markers, mean arterial pressure, and uterine artery Doppler indices are incorporated longitudinally, the same patient may demonstrate a progressively diverging trajectory from expected physiological adaptation between 11 and 20 weeks of gestation. In such a scenario, dynamic updating of risk could shift the estimated probability of disease to 1:40 by mid-pregnancy, crossing a clinically actionable threshold. Importantly, this change would not arise from a single abnormal value, but from the slope of biological change across time. Trajectory-based models therefore offer the possibility of identifying clinically meaningful risk earlier in women whose initial absolute risk appears modest but whose evolving biological profile signals emerging vulnerability.

Longitudinal modeling extends this paradigm by explicitly incorporating the temporal dimension of risk. Preeclampsia does not arise abruptly; rather, it develops along a continuum in which early placental and maternal adaptations shape downstream vulnerability. Repeated measurements of clinical parameters and biomarkers enable characterization of risk trajectories, revealing deviations from expected physiological adaptation rather than isolated abnormal values [25,29,32]. These trajectories may differ substantially across mechanistic phenotypes, with some characterized by early and progressive divergence and others by later or more abrupt changes.

Trajectory-based frameworks offer several conceptual advantages. They facilitate phenotype-aware prediction by aligning evolving biological signals with underlying mechanisms, rather than forcing heterogeneous cases into a single risk category. They also provide a natural framework for updating risk estimates as pregnancy progresses, allowing prediction to remain responsive to new information. This dynamic perspective is particularly relevant in early pregnancy, when absolute risk may initially be low but trajectories may already signal increased vulnerability.

Despite their promise, multimarker and longitudinal approaches introduce important challenges. Increased model complexity raises concerns related to data requirements, missingness, and interpretability, particularly in real-world clinical settings. In addition, the incremental predictive value of additional markers must be carefully weighed against feasibility, cost, and clinical utility. Rigorous validation is therefore essential to ensure that observed trajectories reflect meaningful biological processes rather than noise or overfitting [25,33].

By framing preeclampsia risk as an evolving and multidimensional process, multimarker and longitudinal models offer a conceptual foundation for more precise and clinically relevant prediction. Rather than relying on fixed thresholds, these approaches emphasize how risk emerges and changes across gestation, providing a framework for predictive models that are both biologically informed and dynamically updated.

## 6. Predictive Models: From Classical Statistics to Machine Learning

Predictive modeling has long been central to efforts aimed at identifying women at risk of preeclampsia. Traditional statistical approaches, including logistic regression and survival models, remain the foundation of most clinically implemented prediction tools. These models offer important advantages, such as transparency, interpretability, and the ability to directly estimate absolute risk. When appropriately specified and rigorously validated, classical models provide robust performance and remain highly relevant for clinical decision-making [25,26,33].

At the same time, classical approaches face inherent limitations when applied to complex, high-dimensional biological data. Linear assumptions, prespecified interactions, and limited capacity to capture nonlinear relationships may restrict their ability to fully leverage multimarker information or longitudinal trajectories. These constraints have contributed to increasing interest in machine learning techniques, which can flexibly model complex patterns and interactions without strong a priori assumptions [33,34].

Machine learning methods—including tree-based algorithms, ensemble approaches, and neural networks—have shown promising discriminatory performance in preeclampsia prediction studies, particularly when integrating diverse clinical, biochemical, and imaging inputs [7,8]. In principle, these methods are well suited to capturing nonlinear dynamics and interactions that reflect underlying biological complexity. In practice, however, improvements in discrimination do not necessarily translate into improved clinical utility [26,35].

Several challenges limit the broader adoption of machine learning-based prediction models in preeclampsia. Overfitting remains a major concern, particularly in studies with modest sample sizes relative to model complexity. Apparent gains in performance often fail to generalize beyond the development dataset, underscoring the critical importance of external validation [25,33]. Moreover, many machine learning models operate as “black boxes,” offering limited insight into which features drive predictions or how risk evolves over time. This lack of interpretability poses barriers to clinical trust, regulatory acceptance, and meaningful integration into decision-making [34,35].

An additional limitation is the frequent disconnect between commonly reported performance metrics and clinical relevance. Emphasis is often placed on measures such as the area under the receiver operating characteristic curve, while calibration, absolute risk estimation, and decision-analytic metrics receive comparatively less attention [26,36,37]. Models that perform well in ranking risk may nevertheless provide limited guidance for prevention or surveillance if they fail to generate reliable, context-specific risk estimates.

Efforts to address these challenges increasingly focus on hybrid and interpretable modeling strategies that combine the strengths of classical statistics and machine learning. Approaches such as penalized regression, flexible parametric models, and explainable machine learning seek to balance predictive performance with interpretability and clinical usability [33,34]. When embedded within longitudinal and phenotype-informed frameworks, these strategies hold promise for translating complex data into actionable risk assessment.

Ultimately, the choice of predictive model should be guided not by methodological novelty, but by its capacity to support meaningful clinical decisions. Models that are transparent, well calibrated, externally validated, and aligned with biological understanding are more likely to achieve sustained clinical impact than those optimized solely for discrimination. This perspective reinforces the need to evaluate predictive models within integrated frameworks that account for biological heterogeneity, temporal dynamics, and real-world implementation constraints [25,33,37]. Table 2 summarizes representative predictive approaches and their clinical performance characteristics, while Table 3 provides a conceptual framework integrating their biological, temporal, and modeling dimensions.

Predictive approaches in preeclampsia can be conceptualized as a progression from static, single-pathway models toward integrative and dynamic frameworks that incorporate biological heterogeneity and temporal risk evolution. This framework emphasizes how alignment with underlying molecular mechanisms and longitudinal dynamics may improve clinically actionable prediction.

## 7. Translating Prediction into Clinical Decision-Making

The ultimate value of predictive models for preeclampsia lies not in statistical performance alone, but in their capacity to inform meaningful clinical decisions. Prediction that does not influence management, surveillance strategies, or preventive interventions offers limited benefit, regardless of model sophistication. Bridging the gap between risk estimation and clinical action therefore remains a central challenge for the field.

A key issue in clinical translation is the distinction between relative and absolute risk. Many predictive models emphasize discrimination metrics, such as the area under the receiver operating characteristic curve, which capture the ability to rank individuals by risk but do not directly inform decision thresholds. In contrast, clinical decision-making relies on well-calibrated estimates of absolute risk that can be weighed against the potential benefits and harms of intervention. Without reliable calibration and context-specific risk estimates, even highly discriminative models may offer limited support for individualized care [39,40].

Timing is equally critical. Preventive strategies for preeclampsia, including low-dose aspirin and tailored surveillance, are most effective when initiated early in pregnancy, often before overt clinical manifestations emerge [28,41,42]. Prediction frameworks must therefore identify meaningful risk sufficiently early and allow risk estimates to be updated as pregnancy progresses. Models applied at a single gestational time point may fail to capture evolving risk trajectories and may therefore miss opportunities for prevention.

Linking risk prediction to preventive efficacy also requires explicit consideration of how improved risk stratification modifies treatment impact. Preventive interventions such as low-dose aspirin reduce the relative risk of preterm preeclampsia by a relatively constant proportion, yet the absolute benefit depends directly on baseline risk. Consequently, calibration—not discrimination alone—determines the number needed to treat (NNT), since NNT is determined by the magnitude of absolute risk reduction. Overestimation of risk may expand eligibility and dilute benefit through overtreatment, whereas underestimation may exclude women with substantial preventable risk. Phenotype-informed and dynamically updated models may therefore improve absolute risk estimation, better align intervention thresholds with expected treatment effects, and optimize net clinical benefit as assessed through decision curve analysis. In this context, evaluation of prediction models should extend beyond discrimination to include changes in NNT, proportion treated, preventable cases captured, and net benefit across clinically relevant risk thresholds.

The clinical implications of improved calibration can be illustrated with a conceptual example. Consider a population in which the baseline risk of preterm preeclampsia is approximately 3%. If a screening model identifies a subgroup with an estimated risk of 10%, and aspirin prophylaxis reduces the relative risk by about 60%, the absolute risk reduction would be approximately 6 percentage points, corresponding to a NNT of roughly 17. In contrast, if miscalibration leads the same model to overestimate risk and the true baseline risk of the selected group is only 5%, the absolute risk reduction would fall to about 3 percentage points, increasing the NNT to approximately 33. In other words, identical relative treatment efficacy may translate into substantially different preventive efficiency depending on the accuracy of absolute risk estimation. Improving calibration therefore has direct clinical implications, as more accurate risk estimation can concentrate preventive interventions among women most likely to benefit while reducing unnecessary treatment.

Effective clinical implementation further requires alignment between prediction outputs and actionable care pathways. Risk estimates must be interpretable by clinicians and integrated into existing clinical workflows, such as antenatal screening programs and follow-up protocols [43]. Continuous or trajectory-based risk assessment may facilitate stepwise escalation of care rather than binary decisions based on fixed thresholds. At the same time, such approaches must be carefully designed to avoid information overload and to remain practical in routine clinical settings.

Population context also shapes translation. Models developed in selected or high-resource settings may not perform similarly across diverse populations, particularly in low- and middle-income environments where baseline risk, comorbidities, and access to care differ. External validation and, when necessary, recalibration are therefore essential to ensure that predictive tools remain clinically meaningful across settings [39,40]. Failure to address these issues risks widening disparities rather than improving outcomes.

Finally, successful translation depends on rigorous evaluation of clinical impact. Beyond predictive accuracy, models should be assessed according to their ability to improve decision-making, optimize resource allocation, and ultimately reduce maternal and perinatal morbidity. This requires moving beyond retrospective validation toward prospective implementation studies that explicitly link risk stratification to intervention [44].

Taken together, translating prediction into clinical decision-making requires a shift from model-centered to patient-centered evaluation. Predictive tools that are timely, interpretable, well calibrated, and embedded within clear clinical pathways are more likely to deliver meaningful clinical benefit than those optimized solely for statistical performance.

## 8. Future Directions and Research Priorities

Advancing prediction in preeclampsia will require a shift from incremental refinement of existing tools toward the development of integrative, biologically informed frameworks explicitly designed for clinical translation. Several research priorities emerge from the limitations and opportunities outlined in this review.

First, future work should move beyond the pursuit of isolated predictors and instead prioritize phenotype-aware strategies that align biomarkers and predictive models with underlying biological mechanisms. This approach entails deliberate integration of markers reflecting placental function, maternal cardiovascular adaptation, and inflammatory or metabolic pathways, selected according to gestational timing and hypothesized pathophysiology. Studies that directly test whether phenotype-informed models improve prediction, calibration, or clinical utility compared with generic approaches are particularly needed.

Second, longitudinal data should be treated as a central component of prediction rather than as a secondary enhancement. Repeated measurements of clinical and biological parameters provide unique insight into risk trajectories and disease evolution that cannot be captured by single time-point assessments. Methodological efforts should prioritize models capable of accommodating temporal dynamics, updating risk estimates across gestation, and distinguishing meaningful biological change from random variability [38].

**Figure 3:**
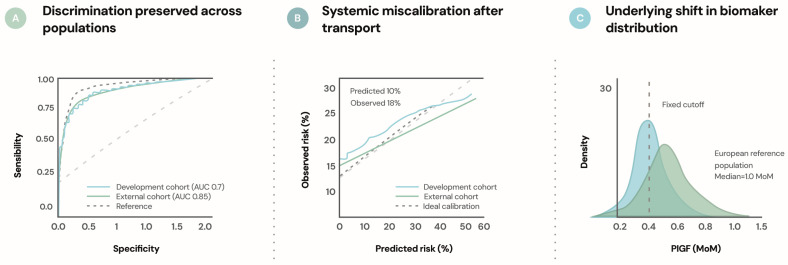
Transportability challenges in prediction models for preeclampsia. This figure represents a conceptual illustration of model performance across populations. (**A**) Discrimination may remain stable across populations even when model transport occurs. (**B**) Despite preserved discrimination, systematic miscalibration may arise when baseline risk differs between populations. (**C**) Differences in biomarker distributions across populations can further contribute to miscalibration when fixed thresholds are applied. These patterns illustrate why transportability testing and recalibration are essential when implementing prediction models across diverse clinical settings.

Third, greater emphasis must be placed on validation, calibration, and transportability. Predictive models should be evaluated across diverse populations and health care settings, with particular attention to recalibration and performance in low- and middle-income contexts. As illustrated in Figure 3, models transported across populations may preserve discrimination while exhibiting systematic miscalibration when baseline risk and biomarker distributions differ. Testing model transportability across populations with different baseline risks and biomarker distributions will be essential to ensure that prediction tools remain clinically meaningful beyond their development cohorts. Reporting standards should extend beyond discrimination metrics to include calibration, absolute risk estimates, and decision-analytic measures that reflect clinical relevance. Transparent reporting of model development and validation remains essential to support reproducibility and trust [45,46,47].

Fourth, interpretability and clinical usability should be considered core design principles rather than optional features. Regardless of whether models are based on classical statistics, machine learning, or hybrid approaches, they must generate outputs that clinicians can understand and act upon. Explainable modeling strategies and explicit links between risk estimates and management pathways will be critical for successful implementation [45,47].

Finally, future studies should increasingly evaluate the clinical impact of prediction. Prospective implementation studies that explicitly link risk stratification to preventive interventions, surveillance strategies, or resource allocation are necessary to demonstrate value beyond predictive accuracy. Prediction research must therefore move beyond statistical performance toward frameworks that explicitly quantify how improved risk stratification modifies preventive efficiency and clinical outcomes. Without evidence of clinical benefit, even well-performing models are unlikely to achieve sustained adoption [48,49].

Collectively, these priorities underscore the need to reframe preeclampsia prediction as an integrative, longitudinal, and clinically anchored endeavor. Progress in this direction has the potential not only to improve risk assessment, but also to support more precise, timely, and equitable care for women at risk of preeclampsia.

## 9. Conclusions

Prediction in preeclampsia appears to be at a critical stage. The limited clinical impact of decades of biomarker discovery and model development likely reflects not a lack of data, but the incomplete integration of biological heterogeneity, temporal dynamics, and clinical context within predictive frameworks. Recognizing preeclampsia as a spectrum of mechanistic phenotypes that evolve across gestation suggests a shift from viewing prediction as a static classification problem toward a more longitudinal and multidimensional process. Multimarker and trajectory-based approaches, supported by transparent, well-calibrated, and externally validated predictive models, may provide a pathway toward more clinically meaningful risk stratification, particularly when aligned with actionable care pathways. Ultimately, progress in preeclampsia prediction will likely depend less on the identification of additional isolated biomarkers and more on the integration of mechanistic insight, dynamic modeling, and implementation science to support tools with the potential to improve prevention, clinical decision-making, and maternal–perinatal outcomes.

## Figures and Tables

**Table 1:** Mechanistic phenotypes of preeclampsia and their clinical implications.

Phenotype	Dominant Pathway	Typical Timing	FGR	Angiogenic Profile	Clinical Implications
Placental–angiogenic phenotype	Impaired placentation and angiogenic imbalance	Predominantly early pregnancy	Frequently present	Markedly altered (increased sFlt-1, decreased PlGF)	High risk of early-onset disease, fetal growth restriction, and adverse perinatal outcomes; well suited for angiogenic biomarker-based risk stratification and short-term prediction
Maternal cardiovascular phenotype	Inadequate maternal cardiovascular adaptation to pregnancy	Predominantly late pregnancy	Usually absent	Normal or mildly altered	Disease driven primarily by maternal hemodynamic vulnerability; angiogenic markers may be less informative, highlighting the need for cardiovascular-focused and longitudinal prediction strategies
Inflammatory–metabolic phenotype	Systemic inflammation, immune dysregulation, and metabolic stress	Across gestation	Variable	Variable or modestly altered	Frequently associated with obesity, diabetes, or metabolic comorbidity; modulates and amplifies other pathways, underscoring the value of integrating metabolic and inflammatory markers
Mixed/overlapping phenotypes	Coexistence of multiple mechanistic pathways	Variable	Variable	Heterogeneous	Represents the majority of real-world cases; challenges single-pathway prediction and supports the need for multimarker, phenotype-aware, and longitudinal frameworks

FGR: fetal growth restriction; sFlt-1: soluble fms-like tyrosine kinase-1; PlGF: placental growth factor.

**Table 2:** Representative predictive approaches in preeclampsia: calibration, validation, and clinical usefulness.

Approach	Representative Example(s)	Calibration	Validation	Clinical Usefulness	Main Limitation
Single-biomarker strategies	sFlt-1/PlGF ratio [9,23,24]	Good for short-term symptomatic prediction; population-dependent for screening	Validated in suspected PE settings	Rule out and short-term triage	Limited for early risk stratification
Multimarker first-trimester screening	FMF screening algorithm [6,27,28,30]	Good in screened populations; may require recalibration	Externally validated in multiple cohorts	Supports early risk stratification and aspirin prophylaxis	Static first-trimester snapshot
Classical regression-based models	Logistic/competing-risk models [25,26,33]	Can be well calibrated when appropriately specified	Variable across cohorts	Absolute risk estimation and structured screening	Model specification and transportability
Machine learning approaches	Multimodal clinical/biochemical models [7,8,14,35]	Often incompletely reported	External validation limited	Potential decision-support role	Limited interpretability and transparency
Longitudinal models	Repeated-measures/trajectory-based models [32,38]	Emerging	Limited but growing	Dynamic risk updating across gestation	Data intensity and implementation complexity
Phenotype-informed models	Mechanistic-domain models [10,11,16]	Early-stage	Limited validation	Precision-oriented risk stratification	Phenotype definition and validation remain needed

This table summarizes representative predictive approaches in preeclampsia, highlighting their calibration, validation context, clinical usefulness, and key limitations relevant to clinical application.

**Table 3:** Conceptual framework for the evolution of predictive strategies in preeclampsia.

Approach	Biological Basis	Temporal Representation	Model Perspective	Clinical Implication
Single-marker strategies	Single dominant pathway (e.g., angiogenic imbalance)	Static	Threshold-based	Short-term risk assessment; limited early prediction
Multimarker strategies	Partial integration of multiple pathways	Static	Additive/combined signals	Improved discrimination; limited adaptability
Regression-based models	Selected predictors reflecting clinical and biological factors	Static	Risk estimation	Provides absolute risk; dependent on model specification
Machine learning approaches	Complex multi-pathway interactions	Mostly static	Pattern recognition	High discrimination; limited interpretability and transportability
Longitudinal models	Evolving biological processes across gestation	Dynamic	Trajectory-based	Enables dynamic risk updating and earlier detection
Phenotype-informed models	Mechanistically defined and interacting biological domains	Dynamic	Biology-driven	Aligns prediction with disease mechanisms; supports personalized prevention

## Data Availability

The original contributions presented in this study are included in the article. Further inquiries can be directed to the corresponding author.
